# An Integrated Multidimensional Framework for Hospital Performance Assessment: Design and Application in Romanian Public Hospitals

**DOI:** 10.3390/healthcare14172671

**Published:** 2026-08-22

**Authors:** Cristina Elena Dobre, Robert Daniel Negru, Doina Clementina Cojocaru, Eliza Maria Fotache, Ioana Rudnic, Liliana Sachelarie, Alina Elena Jehac, Liviu Stafie

**Affiliations:** 1 “Socola” Institute of Psychiatry, Bucium Road 36, 700282 Iași, Romania; cedobre@yahoo.com; 2 Department of Internal Medicine, “Grigore T. Popa” University of Medicine and Pharmacy, 700115 Iași, Romania; robert.negru@umfiasi.ro (R.D.N.); liviu.stafie@dspiasi.ro (L.S.); 3 Department of Medical Specialties I, “Grigore T. Popa” University of Medicine and Pharmacy, 700115 Iași, Romania; doina.cojocaru@umfiasi.ro; 4 Department of Dental Medicine, “Grigore T. Popa” University of Medicine and Pharmacy, 700115 Iași, Romania; ioana.rudnic@umfiasi.ro (I.R.); alina.jehac@umfiasi.ro (A.E.J.); 5 Department of Dental Medicine, Apollonia University, 700511 Iași, Romania

**Keywords:** hospital performance, multidimensional assessment, organizational management, healthcare quality, patient satisfaction, cost efficiency, Stochastic Frontier Analysis, public hospitals, healthcare management

## Abstract

Background/Objectives: Hospital performance is a multidimensional construct that cannot be adequately assessed using isolated financial, clinical, organizational, or patient-reported indicators. This study aimed to develop and evaluate an Integrated Multidimensional Hospital Performance Assessment Framework combining organizational management, healthcare quality, patient satisfaction, and cost efficiency. Methods: An observational, descriptive, comparative study was conducted using data collected during 2018–2019 from two Romanian public hospitals with different organizational profiles: a multidisciplinary general hospital and a monospecialty hospital. Organizational, operational, financial, and clinical indicators were analyzed comparatively. Patient satisfaction was assessed in 184 hospitalized patients, including 130 from the general hospital and 54 from the monospecialty hospital, using a structured questionnaire with excellent internal consistency (Cronbach’s α = 0.92). Cost efficiency was estimated using Stochastic Frontier Analysis based on hospital expenditure, activity, resource utilization, case complexity, and quality-related variables. Results: Significant differences were identified between the hospitals in staff composition, bed occupancy, outpatient activity, case-mix complexity, surgical activity, healthcare quality, and financial performance. Patient satisfaction was generally high in both institutions, although several dimensions differed significantly, particularly overall satisfaction, waiting time, physician consultation, treatment received, and adherence to the prescribed medication schedule. The general hospital had a lower mean cost-inefficiency score than the monospecialty hospital (0.253 vs. 0.395), corresponding to higher estimated cost efficiency (0.747 vs. 0.605; *p* < 0.001). Conclusions: The proposed framework offers a comprehensive and practical approach to hospital performance assessment, and its application in two public hospitals provides a proof-of-concept demonstration of feasibility. Further multicenter studies are required for external validation and assessment of its generalizability across different hospital settings.

## 1. Introduction

Hospitals represent the core of healthcare systems, delivering specialized medical services while balancing increasing demands for high-quality care, patient safety, financial sustainability, and efficient resource utilization. Demographic aging, the rising prevalence of chronic diseases, technological innovation, workforce shortages, and escalating healthcare expenditures have increased the complexity of hospital management. Consequently, improving hospital performance has become a strategic priority worldwide, requiring robust evaluation methodologies to support evidence-based managerial and policy decisions [1,2,3,4].

Hospital performance is a multidimensional construct that cannot be adequately described by a single indicator or domain. Traditional assessment has relied on financial indicators, productivity measures, clinical outcomes, or operational statistics, which provide valuable but partial information on hospital functioning. Modern performance assessment therefore requires approaches that consider organizational management, healthcare quality, patient-centered care, and resource efficiency together [5,6,7,8].

Healthcare quality assessment has similarly expanded beyond clinical effectiveness and patient safety to include accessibility, continuity of care, organizational responsiveness, patient experience, and healthcare outcomes. Patient-centered care and continuous quality improvement are consequently recognized as important components of hospital performance assessment [9,10,11].

At the same time, healthcare managers face increasing pressure to optimize available resources without compromising quality of care. Frontier efficiency methods, particularly Data Envelopment Analysis (DEA) and Stochastic Frontier Analysis (SFA), have therefore been increasingly applied to hospital performance evaluation. SFA offers the advantage of separating random statistical variation from cost inefficiency and accounting for measurement error, allowing efficiency to be evaluated under real-world healthcare conditions [12,13,14,15].

Despite these developments, hospital performance dimensions are frequently examined separately. Cost-efficiency studies generally emphasize economic and operational performance, quality studies focus primarily on clinical indicators, while patient satisfaction research evaluates communication, accessibility, interpersonal relationships, hospital environment, and patient experience. Such separate analyses may provide only a partial representation of institutional performance and limit their usefulness for managerial decision-making [16,17,18,19,20].

Previous multidimensional approaches have sought to combine organizational, clinical, financial, and patient-centered dimensions within broader hospital performance assessment models. Such approaches may support benchmarking, identification of institutional strengths and weaknesses, quality improvement, and healthcare planning [21,22,23,24]. However, the joint assessment of organizational management, healthcare quality, patient satisfaction, and cost efficiency estimated through Stochastic Frontier Analysis within a single hospital-level application remains less frequently explored.

In Romania, hospital performance assessment continues to rely predominantly on administrative and financial indicators reported within the national healthcare system. Although quality indicators and patient satisfaction surveys have gained importance, these dimensions are generally interpreted separately and are rarely considered together with efficiency analyses. This limits the availability of multidimensional tools supporting hospital-level management and performance evaluation [25,26,27].

To address this methodological gap, the present study proposes an Integrated Multidimensional Hospital Performance Assessment Framework combining four complementary dimensions: organizational management, healthcare quality, patient satisfaction, and cost efficiency estimated through SFA. The contribution of the proposed framework lies not in introducing multidimensional performance assessment itself, but in the structured joint interpretation of these four domains, including an econometric efficiency component, within a single hospital-level application.

The framework was developed and applied as a proof-of-concept using data from two Romanian public hospitals representing different organizational models of healthcare delivery. The application was intended to assess the feasibility and practical applicability of the proposed approach rather than to establish external validation. By jointly considering organizational, clinical, economic, and patient-centered information, the study explores the potential utility of the framework for hospital performance assessment, while further multicenter studies are required to establish its generalizability across healthcare settings [28,29,30].

Although previous multidimensional hospital performance frameworks have combined organizational, clinical, financial, or patient-centered indicators, these domains are frequently assessed using parallel sets of indicators rather than being integrated with an econometric measure of resource efficiency within a unified decision-support structure (Table 1).

In particular, the simultaneous interpretation of organizational management, healthcare quality, patient-reported satisfaction, and cost efficiency estimated through Stochastic Frontier Analysis remains insufficiently addressed. The proposed framework seeks to bridge this methodological gap by integrating these four complementary domains within a single analytical structure and translating their combined assessment into actionable information for strategic planning, resource allocation, quality improvement, and performance monitoring. Its contribution therefore lies not in introducing new individual performance indicators, but in their structured integration with an econometric efficiency component and their joint interpretation for hospital-level managerial decision-making.

## 2. Materials and Methods

### 2.1. Study Design

This study was designed as an observational, descriptive, and comparative study to develop and apply an Integrated Multidimensional Hospital Performance Assessment Framework for public healthcare institutions. The proposed framework combines organizational management, healthcare quality, patient satisfaction, and cost efficiency into a unified methodological model for hospital performance evaluation.

The comparative analysis was conducted using data collected from two Romanian public hospitals representing different organizational models of healthcare delivery: a multidisciplinary general hospital and a monospecialty hospital. The study covered a continuous two-year observation period (2018–2019), enabling the assessment of organizational performance under routine clinical and managerial conditions.

Unlike conventional hospital evaluation models that investigate individual dimensions separately, the present methodology integrates administrative, organizational, financial, clinical, and patient-reported indicators within a single analytical framework. This multidimensional approach was designed to provide a comprehensive assessment of hospital performance and to facilitate evidence-based managerial decision-making.

### 2.2. Study Setting

The study was conducted in two Romanian public hospitals that operate within the national health insurance system. The study was conducted in accordance with the ethical principles of the Declaration of Helsinki and applicable regulations governing research involving human participants. Ethical approval for the doctoral research protocol was granted by the Research Ethics Committee of Nicolae Testemițanu State University of Medicine and Pharmacy (Protocol No. 63, 18 June 2015). To preserve institutional confidentiality, the participating hospitals are presented according to their organizational profile rather than by institutional name. One hospital represents a multidisciplinary general hospital providing medical and surgical services across multiple specialties, whereas the second represents a monospecialty hospital focused on a single medical specialty. Both institutions deliver secondary and tertiary healthcare services under comparable legislative and financing regulations.

The comparative design was selected to evaluate hospital performance across organizations characterized by different management structures, clinical complexity, resource allocation patterns, service utilization, and organizational objectives. Although both institutions function within the same national healthcare system, differences in organizational structure and service delivery provide an appropriate setting for the proof-of-concept application of the proposed multidimensional performance assessment framework. Hospital activity was evaluated using routinely collected institutional data obtained during the study period. Information included organizational, operational, financial, and clinical indicators together with patient satisfaction data collected through standardized questionnaires administered during hospitalization. All datasets were processed using identical methods to ensure comparability across hospitals.

### 2.3. Integrated Multidimensional Hospital Performance Assessment Framework

Hospital performance was assessed using an integrated framework comprising four complementary domains: organizational management, healthcare quality, patient satisfaction, and cost efficiency. This approach was based on the premise that hospital performance cannot be adequately evaluated using isolated indicators.

Organizational management was assessed through indicators of human, structural, financial, and operational resource use. Healthcare quality included routinely monitored clinical outcomes and quality-of-care indicators. Patient satisfaction was evaluated using standardized questionnaires addressing communication, accessibility, hospital services, and overall experience. Cost efficiency was estimated through Stochastic Frontier Analysis (SFA), which examined the relationship between hospital resources and outputs while accounting for random variation and inefficiency.

The four dimensions are integrated through the joint interpretation of their complementary results within a common performance assessment structure. Organizational management reflects institutional capacity and resource use, healthcare quality captures clinical performance and patient safety, patient satisfaction incorporates the patient perspective, and cost efficiency evaluates resource utilization. Together, these dimensions provide an overall assessment of hospital performance and allow the identification of institutional strengths, weaknesses, and priority areas for improvement.

**Figure 1:**
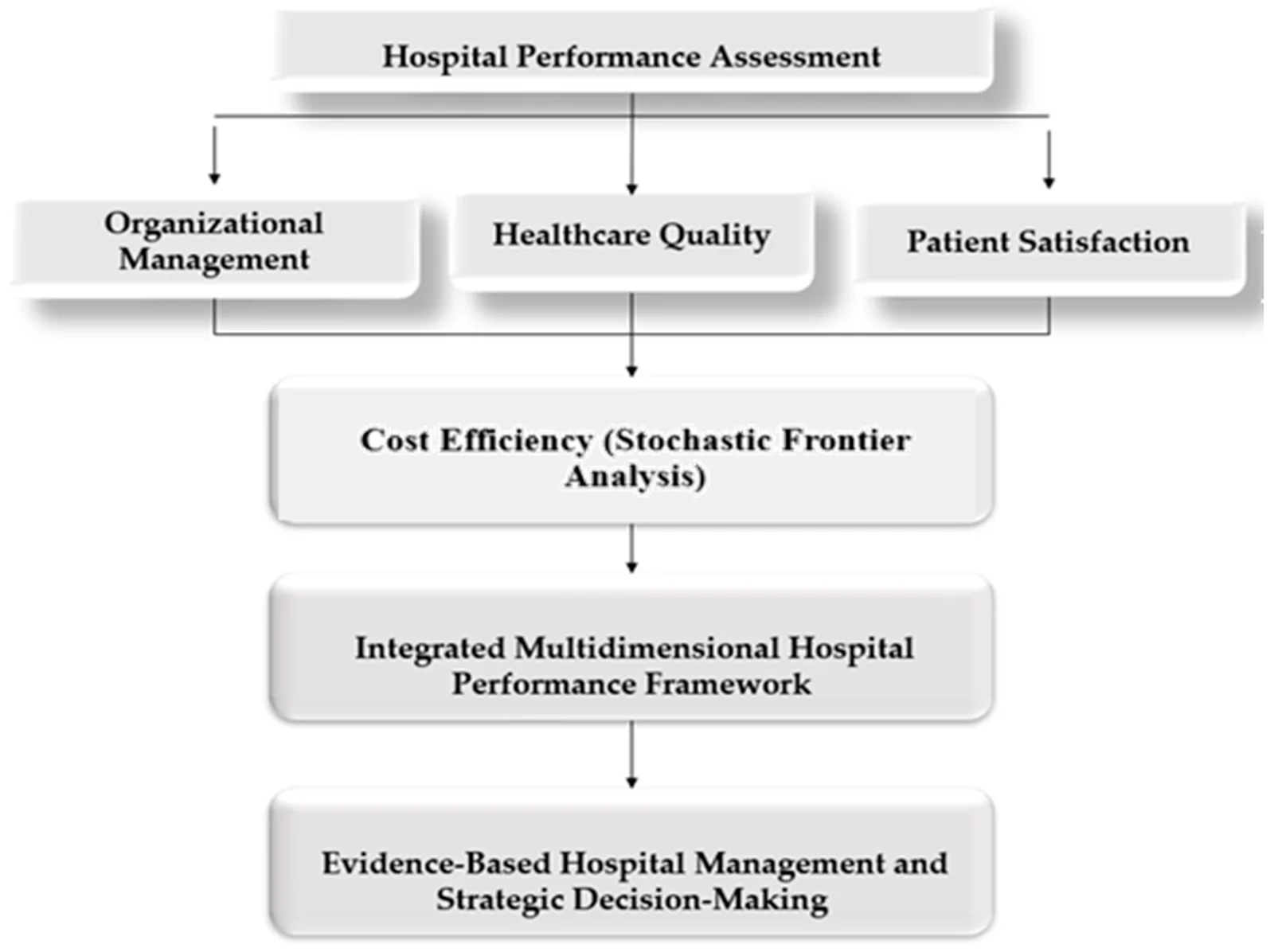
Conceptual structure of the proposed Integrated Multidimensional Hospital Performance Assessment Framework.

The framework structure is presented in Figure 1.

Integration within the proposed framework was conceptual rather than based on mathematical aggregation. The four domains were assessed using their domain-specific indicators and analytical methods and were subsequently interpreted jointly within a common decision-support structure. No weighting scheme, cross-domain normalization, or aggregation algorithm was applied, and the framework did not generate a single composite performance score. Accordingly, integration refers to the structured joint interpretation of complementary performance dimensions rather than their reduction to a single numerical index.

### 2.4. Hospital Performance Indicators

Hospital performance was assessed using a comprehensive set of routinely collected indicators, grouped into four major categories based on their functional roles within the proposed multidimensional framework. Indicator selection was based on their relevance to hospital management, availability in institutional databases, and applicability to comparative performance assessment.

Indicator selection was theoretically guided by the multidimensional concept of hospital performance, according to which institutional performance reflects the interaction between organizational capacity, healthcare quality, resource utilization, financial sustainability, and patient experience [2,4,9,20,21]. Empirically, indicators were retained when they were routinely collected in both participating hospitals, consistently available throughout the study period, comparable across the two institutions, and directly relevant to hospital activity, quality of care, resource use, or patient experience. This combined theoretical and empirical selection strategy was intended to ensure both conceptual relevance and practical applicability of the indicators within the proposed framework.

The first category included organizational and operational indicators, reflecting hospital capacity, human resource allocation, service utilization, and healthcare activity. These indicators included the number of hospital beds, medical personnel, nursing staff, and auxiliary personnel; the average length of hospital stay; the bed occupancy rate; the number of discharged patients; outpatient consultations; emergency admissions; and surgical activity.

The second category consisted of clinical quality indicators, selected to evaluate healthcare quality and patient safety. These variables included hospital mortality, healthcare-associated infections, readmission rates, diagnostic concordance, and other quality indicators routinely monitored by hospital management as part of institutional quality assurance programs.

The third category included financial indicators, which evaluated hospital resource utilization and economic performance. The analyzed variables comprised total hospital expenditures, personnel costs, pharmaceutical expenditures, budget execution, own-source revenues, hospitalization costs, and average cost per patient, allowing the assessment of financial sustainability and resource allocation efficiency.

The fourth category incorporated patient-centered indicators, obtained through standardized patient satisfaction questionnaires. These variables measured patients’ perceptions regarding access to healthcare, communication with healthcare professionals, information provided during hospitalization, hospital environment, accommodation conditions, respect for patients’ rights, and overall satisfaction with healthcare services.

Grouping indicators into these four domains allowed each component of hospital performance to be evaluated independently while simultaneously supporting their integration within the proposed multidimensional assessment framework.

### 2.5. Patient Satisfaction Assessment

Patient satisfaction was assessed using a structured questionnaire specifically developed to evaluate patients’ perceptions of the healthcare services provided by the participating hospitals (Table S1). The questionnaire addressed communication with healthcare professionals, access to medical services, information provided during hospitalization, respect for patients’ rights, hospital environment, treatment received, and overall satisfaction with healthcare services.

Before its implementation, the questionnaire underwent a validation process using a pre-test sample of 30 hospitalized patients. Items showing poor statistical relevance or redundancy were removed during the validation procedure. Internal consistency was assessed using Cronbach’s alpha, which indicated excellent reliability (α = 0.92).

The final study included 184 hospitalized patients, comprising 130 patients from the general hospital and 54 patients from the monospecialty hospital. Participants were selected using random sampling. Inclusion and exclusion criteria were established to ensure a representative study population and to include only respondents able to understand and complete the questionnaire.

Participants completed the questionnaires anonymously, and all responses were processed confidentially before statistical analysis. Patient satisfaction data were subsequently integrated with organizational management, healthcare quality, and cost efficiency indicators within the proposed multidimensional hospital performance assessment framework.

### 2.6. Cost Efficiency Assessment Using Stochastic Frontier Analysis

Cost efficiency was assessed using Stochastic Frontier Analysis (SFA), an econometric approach that estimates the minimum cost required to deliver a given level of hospital services while distinguishing random variation from cost inefficiency. Compared with deterministic methods, SFA accounts for statistical noise arising from factors beyond managerial control, making it particularly suitable for evaluating hospital performance under heterogeneous organizational and clinical conditions.

The stochastic cost frontier was specified using total hospital expenditure (excluding investment costs) as the dependent variable and was estimated using Maximum Likelihood Estimation (MLE). The inefficiency component was assumed to follow a half-normal distribution, while the random error component was assumed to be normally distributed. The model was specified as a random-effects regression model to account for repeated hospital-level observations.

The explanatory variables included the number of discharged patients, outpatient consultations, the interaction between discharged patients and outpatient consultations, average length of hospital stay, case-mix index, proportion of surgical patients, healthcare-associated infection rate, readmission rate, employee-to-bed ratio, bed occupancy rate, and total number of hospital beds. These variables were selected to represent complementary dimensions of hospital activity, resource utilization, service complexity, and healthcare quality, while ensuring the use of routinely collected institutional data available for both participating hospitals.

The analysis generated cost-inefficiency scores representing the proportional deviation between observed hospital costs and the minimum feasible costs estimated by the stochastic frontier model. Lower cost-inefficiency scores indicated higher levels of cost efficiency. The resulting cost-efficiency estimates constituted one of the four domains of the proposed Integrated Multidimensional Hospital Performance Assessment Framework and were interpreted together with organizational management, healthcare quality, and patient satisfaction indicators.

### 2.7. Statistical Analysis

Statistical analyses were performed using IBM SPSS Statistics version 29.0 (IBM Corp., Armonk, NY, USA), whereas the Stochastic Frontier Analysis (SFA) was implemented in Python version 3.11. Descriptive statistics were calculated for all study variables and are presented as means ± standard deviations (SD) for continuous variables and as absolute frequencies and percentages for categorical variables.

Comparisons between general and monospecialty hospitals were performed using statistical tests appropriate to the data type and distribution. Associations between categorical variables were evaluated using the Chi-square (χ^2^) test; for patient satisfaction comparisons, *p*-values were obtained using Monte Carlo simulations of the Pearson Chi-square test. Differences between continuous variables were assessed using one-way analysis of variance (ANOVA). For comparisons involving two groups, one-way ANOVA provides an equivalent test of differences in group means to the independent-samples *t*-test. The internal consistency and reliability of the patient satisfaction questionnaire were evaluated using Cronbach’s alpha coefficient. Given the exploratory nature of the patient satisfaction comparisons, no formal adjustment for multiple testing was applied, and individual *p*-values were interpreted cautiously.

For the SFA, 48 hospital-month observations were included, corresponding to monthly observations from the two participating hospitals over the 24-month study period (January 2018–December 2019). Each hospital contributed 24 repeated monthly observations, with the hospital-month representing the unit of analysis. Total hospital expenditure (excluding investment costs) served as the dependent variable, while organizational, clinical, and operational indicators were included as explanatory variables. Model parameters were estimated using Maximum Likelihood Estimation (MLE), enabling estimation of cost inefficiency while accounting for random variation in hospital performance. The statistical significance of the difference in mean cost-inefficiency scores between the general and monospecialty hospitals was assessed using a two-sample *t*-test with unequal variances.

Given that the 48 hospital-month observations were derived from only two institutions and that several explanatory variables were included in the SFA specification, the resulting parameter and cost-inefficiency estimates should be interpreted with caution. The repeated monthly observations provide longitudinal information within each hospital but do not compensate for the limited number of institutions; therefore, the SFA estimates are considered exploratory rather than robust estimates intended for generalization across hospitals.

A two-sided *p*-value < 0.05 was considered statistically significant at the 95% confidence level.

## 3. Results

### 3.1. Organizational Management Indicators

The organizational management domain included indicators related to human resources, hospital activity, healthcare quality, and financial performance. Comparative analyses between the general hospital and the monospecialty hospital identified significant differences across several operational indicators.

#### Human Resources

The comparative analysis of human resources revealed significant differences in staff composition between the two hospitals (Table 2). The proportion of medical personnel was significantly higher in the general hospital (92.45%) than in the monospecialty hospital (91.66%; *p* = 0.034). Conversely, the proportion of administrative staff (TESA) was significantly greater in the monospecialty hospital (4.66% vs. 3.66%; *p* = 0.0016). No statistically significant difference was observed in the proportion of support personnel (*p* = 0.056).

Overall, the findings indicate differences in the distribution of healthcare personnel between the two hospitals, whereas the monospecialty hospital allocated a greater proportion of its workforce to administrative activities.

### 3.2. Comparative Analysis of Patient Satisfaction

Patient satisfaction assessment revealed generally high levels of satisfaction in both hospitals across most evaluated domains. Statistically significant differences were observed for several aspects of healthcare delivery, indicating variations in patients’ perceptions of the services provided by the two institutions.

The highest satisfaction scores were associated with the professionalism of healthcare personnel, communication with physicians and nurses, and the quality of medical care. Conversely, lower satisfaction levels were observed for hospital accommodation, waiting times, and selected administrative aspects of healthcare delivery. Comparative analysis demonstrated that several patient-reported indicators differed significantly between the general hospital and the monospecialty hospital, reflecting differences in organizational structure and service delivery.

The results indicate that patient satisfaction represents an essential component of hospital performance, complementing traditional organizational and clinical indicators by providing direct information regarding patients’ experiences and perceived quality of care. The detailed comparative results are presented in Table 3.

### 3.3. Cost Efficiency Analysis (SFA)

Cost efficiency was evaluated using Stochastic Frontier Analysis (SFA), and the resulting cost-inefficiency scores revealed measurable differences in resource utilization between the two hospitals. Lower inefficiency scores indicated better cost efficiency, reflecting hospital performance relative to the estimated stochastic cost frontier.

Although both institutions operated within the same Romanian public healthcare system, their cost-efficiency profiles differed.

Given the limited number of institutions included in the analysis, these SFA estimates should be interpreted with caution as exploratory evidence in the present proof-of-concept application.

The general hospital demonstrated a lower mean cost-inefficiency score (0.253) than the monospecialty hospital (0.395), indicating a higher level of cost efficiency. These differences may be associated with variations in service complexity, organizational structure, patient case-mix, and resource allocation.

The SFA findings complemented the organizational, quality, and patient satisfaction indicators by providing an economic perspective on hospital performance. Together, these results support the multidimensional nature of the proposed assessment framework, showing that hospital performance cannot be adequately characterized using a single category of indicators.

The cost-efficiency results for the two hospitals are presented in Table 4.

### 3.4. Integrated Hospital Performance Assessment

The integration of organizational management, healthcare quality, patient satisfaction, and cost efficiency provided a comprehensive evaluation of hospital performance that could not be achieved using isolated indicators alone.

The general hospital demonstrated superior performance for several organizational and activity-related indicators, including bed occupancy, case-mix complexity, outpatient activity, and financial sustainability. In contrast, the monospecialty hospital showed favorable results for selected clinical outcomes and operational characteristics. Patient satisfaction findings complemented these objective performance indicators by incorporating patients’ perspectives into the overall assessment, whereas SFA quantified the efficiency with which available resources were converted into healthcare services.

The integrated framework demonstrated that hospital performance emerges from the interaction between organizational, clinical, economic, and patient-centered dimensions rather than from a single performance indicator. This multidimensional approach provides decision-makers with a more balanced and evidence-based assessment of institutional performance, facilitating the identification of organizational strengths and opportunities for improvement.

**Figure 2:**
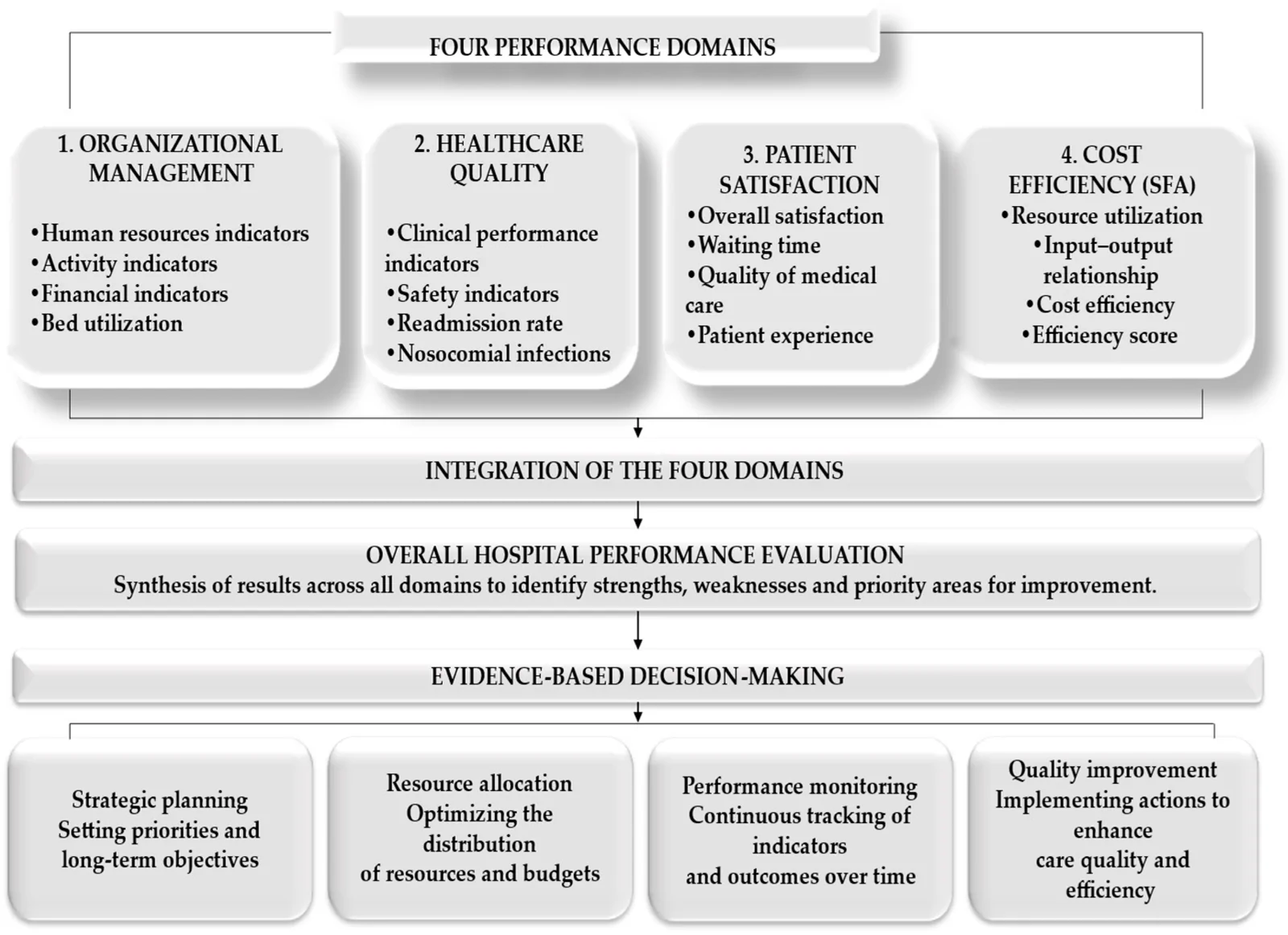
Conceptual integration of hospital performance domains.

The overall integration of the four performance domains is illustrated in Figure 2.

## 4. Discussion

Hospital performance assessment has become increasingly important in modern healthcare systems as hospitals are expected to provide high-quality care while ensuring efficient use of available resources. Consequently, performance evaluation has evolved from traditional approaches based primarily on financial or activity indicators toward multidimensional assessment models that integrate organizational, clinical, patient-centered, and efficiency-related outcomes [13,14,15]. In this context, the present study proposed and applied an integrated hospital performance assessment framework that combines organizational management, healthcare quality, patient satisfaction, and cost efficiency into a single evaluation model. The application of this framework to two Romanian public hospitals with different organizational profiles indicated that hospital performance cannot be adequately interpreted using isolated indicators but rather requires simultaneous analysis of multiple complementary dimensions [16].

Similar observations have been reported by previous investigations, suggesting that organizational performance is associated with resource availability, managerial practices, institutional complexity, and service delivery [17,18,19]. Indicators such as staffing levels, bed occupancy, case-mix index, hospitalization costs, and financial sustainability should therefore be interpreted collectively rather than as independent aspects of hospital activity. Our findings support this multidimensional perspective and the value of joint organizational assessment [20].

Healthcare quality represents another essential component of hospital performance, as it provides information on the effectiveness and safety of medical services. Consistent with previous reports, the present findings support the relevance of quality indicators for assessing institutional performance and clinical effectiveness [21,22]. These indicators have become fundamental components of hospital accreditation systems and quality improvement programs worldwide, emphasizing the importance of continuous monitoring and evidence-based management strategies. The results obtained in the present study reinforce the concept that organizational performance and healthcare quality should be interpreted as complementary dimensions within an integrated evaluation framework.

Patient satisfaction has progressively evolved into one of the most important patient-reported outcome measures and is currently recognized as a key indicator of healthcare quality. Unlike administrative or clinical indicators, patient satisfaction reflects the individual experience of healthcare delivery, encompassing communication with healthcare professionals, responsiveness, accessibility, and perceived quality of medical care [23,24]. The reliability of the assessment instrument and the observed between-hospital differences support the relevance of incorporating patients’ perspectives into institutional evaluation. These findings are consistent with previous studies reporting that improved patient satisfaction is associated with better communication, greater adherence to therapeutic recommendations, increased trust in healthcare professionals, and overall improvement of healthcare outcomes [25].

An important strength of the proposed framework is the integration of cost efficiency assessment using Stochastic Frontier Analysis. While conventional performance indicators quantify healthcare activity or clinical outcomes, Stochastic Frontier Analysis evaluates the efficiency with which available resources are transformed into healthcare services by estimating the distance between observed performance and the stochastic cost frontier [26,27]. The efficiency estimates obtained in the present study suggest that differences in institutional performance are associated not only with the amount of available resources but also with organizational structure, managerial effectiveness, and case complexity. Importantly, the observed efficiency gap between the two hospitals should not be attributed exclusively to managerial performance. Differences in hospital specialization, service volume, patient case-mix, and complexity of care may also contribute to the observed variation in cost efficiency. Although several of these characteristics were incorporated into the SFA specification, their effects cannot be completely separated from the broader organizational context of the two institutions. Therefore, the observed efficiency differences should be interpreted as reflecting a combination of managerial, structural, and case-mix-related factors.

Similar findings have been reported in previous studies applying Stochastic Frontier Analysis and Data Envelopment Analysis to hospital performance assessment, highlighting the value of efficiency analysis as an objective instrument for benchmarking healthcare institutions and identifying opportunities for organizational improvement [28].

Comparison with previously published models demonstrates that hospital performance has traditionally been evaluated through separate analyses of organizational management, healthcare quality, patient satisfaction, or cost efficiency. Although each of these domains provides valuable information, their independent interpretation offers only a partial understanding of institutional performance. The present study extends existing knowledge by integrating these complementary dimensions within a unified conceptual framework, allowing a more comprehensive evaluation of hospital functioning. This integrated approach facilitates the joint assessment of observed patterns and associations across managerial performance, quality of care, patient experience, and cost efficiency, without implying causal relationships between these dimensions [29].

Unlike most previously published approaches, which primarily focus on individual performance dimensions, the proposed model simultaneously evaluates managerial, clinical, patient-centered, and efficiency-related aspects of hospital activity. Consequently, the framework provides a more comprehensive representation of institutional performance and offers healthcare managers an effective tool for strategic planning, quality improvement, performance monitoring, and resource optimization. The contribution of the present study lies in combining organizational, clinical, patient-centered, and SFA-based efficiency assessment within a single application in the Romanian public hospital context [30].

From a practical perspective, the proposed framework may assist hospital managers in jointly identifying organizational, quality, patient-centered, and cost-efficiency areas requiring attention. Its application may support the identification of discrepancies between resource use and performance, facilitate the prioritization of quality-improvement actions, and provide complementary information for resource allocation and strategic planning. Repeated assessment may also allow managers to monitor changes in performance indicators over time and identify areas requiring further organizational review. Furthermore, the proof-of-concept application suggests that the framework may have future potential for benchmarking hospitals with different organizational characteristics. However, its suitability for interinstitutional benchmarking or standardized national performance assessment requires external validation in larger multicenter samples.

Several limitations should be considered when interpreting the present findings. The study included only two Romanian public hospitals, resulting in limited statistical power at the institutional level, limiting the generalizability of the findings, and precluding external validation of the proposed framework. Accordingly, the present application should be regarded as a proof-of-concept demonstrating feasibility rather than as validation of the framework’s robustness or benchmarking performance across institutions. In addition, the retrospective design does not permit causal inference regarding the observed relationships between organizational characteristics and institutional performance. The use of data collected during 2018–2019 may also limit the current applicability of the findings, as hospital organization and healthcare delivery may have evolved since the study period. Furthermore, although the proposed framework integrates four major performance domains, future studies could expand the model by incorporating additional indicators related to digital transformation, healthcare sustainability, organizational resilience, clinical outcomes, and artificial intelligence applications in healthcare management.

Despite these limitations, the present study provides a methodological framework for multidimensional hospital performance assessment and demonstrates the feasibility of its application in two real-world hospital settings. Future multicenter studies involving larger, more diverse hospital populations are warranted to externally validate the proposed framework and evaluate its applicability across healthcare systems. Moreover, integrating advanced analytical approaches, including artificial intelligence, machine learning, and real-time hospital performance monitoring, may further enhance the framework’s capacity to support continuous quality improvement and evidence-based healthcare management [30].

## 5. Conclusions

The present study developed and applied, as a proof-of-concept, an integrated multidimensional framework for hospital performance assessment by combining organizational management, healthcare quality, patient satisfaction, and cost efficiency. The findings indicate that hospital performance cannot be adequately evaluated using isolated indicators, as a comprehensive assessment requires the simultaneous interpretation of managerial, clinical, patient-centered, and efficiency-related dimensions.

The proposed framework offers a practical approach for identifying institutional strengths and weaknesses, supporting resource allocation, quality improvement, and strategic decision-making. The present study represents a proof-of-concept application, and further multicenter studies are needed for external validation across different hospital types and healthcare systems.

## Figures and Tables

**Table 1:** Comparison of representative approaches to hospital performance assessment and the methodological contribution of the present study.

Approach/References	Main Dimensions/Indicators	Method and Data	Limitation/Methodological Positioning
Quality-of-care frameworks [9,20]	Structure, process, outcomes; clinical quality indicators	Conceptual and indicator-based assessment	Limited integration of efficiency and patient-reported outcomes
Hospital efficiency approaches [16,17,18,19]	Resources, costs, activity, outputs	DEA, SFA; hospital administrative/economic data	Limited integration of clinical and patient-reported dimensions
Multidimensional/health-system performance approaches [21,29,30]	Organizational, clinical, financial, patient-centered and value-related dimensions	Multidimensional conceptual and empirical approaches; heterogeneous healthcare datasets	Heterogeneous methods and limited integration of econometric efficiency
Present study	Organizational management, healthcare quality, patient satisfaction, cost efficiency	Comparative analysis + SFA; institutional data from two public hospitals and 184 patients	Integrated four-domain framework combining multidimensional assessment with SFA

**Table 2:** Comparative organizational management indicators of the two hospitals.

Domain	Indicator	General Hospital	Monospecialty Hospital	*p*-Value
Human resources	Medical personnel (%)	92.45	91.66	0.034
	Support personnel (%)	3.90	3.64	0.056
	Administrative personnel (TESA, %)	3.66	4.66	0.002
Hospital activity	Average length of stay (days)	6.36	6.35	0.052
	Bed occupancy rate (%)	78.68	65.37	0.003
	Outpatient consultations, mean	264,526	9010.5	<0.001
	Case-mix index relative to the national level	1.3169	0.8639	0.002
	Surgical operability index (%)	86.81	67.84	0.001
Healthcare quality	In-hospital mortality rate (%)	2.18	0.18	<0.001
	Healthcare-associated infection rate (%)	2.19	0.61	0.001
	Unplanned 30-day readmission rate (%)	2.59	1.90	<0.001
	Admission–discharge diagnostic concordance (%)	87.12	39.84	<0.001
Financial indicators	Budget execution relative to the approved budget (%)	93.24	80.99	0.015
	Own revenues as a proportion of total revenues (%)	42.66	7.02	<0.001
	Continuous hospitalization expenditure as a proportion of total expenditure (%)	83.11	89.80	0.029
	Personnel expenditure as a proportion of total expenditure (%)	58.88	77.73	0.011

Note: Values represent the mean over the two-year study period. Exact *p*-values were rounded to three decimal places; values below 0.001 are reported as *p* < 0.001.

**Table 3:** Comparison of patient satisfaction indicators between the two hospitals.

Patient Satisfaction Indicator	General Hospital, % (95% CI)	Monospecialty Hospital, % (95% CI)	*p*-Value
Overall satisfaction with the quality of medical services	85.4 (79.3–91.5)	77.8 (66.7–88.9)	0.028
Respect for patients’ rights	80.0 (73.1–86.9)	74.1 (62.4–85.8)	0.096
Waiting time until the first medical examination	76.2 (68.8–83.5)	70.4 (58.2–82.5)	0.043
Consultation provided by the attending physician	88.5 (83.0–94.0)	81.5 (71.1–91.8)	0.024
Treatment received during hospitalization	86.2 (80.2–92.1)	74.1 (62.4–85.8)	0.020
Respect and attention provided by the physician	85.4 (79.3–91.5)	85.2 (75.7–94.7)	0.045
Physician’s responsiveness to patients’ questions	83.1 (76.6–89.5)	79.6 (68.9–90.4)	0.027
Physician’s careful management of the patient’s case	86.2 (80.2–92.1)	79.6 (68.9–90.4)	0.014
Adherence to the prescribed medication schedule	91.5 (86.8–96.3)	79.6 (68.9–90.4)	0.003

Note: Values represent the proportion of respondents selecting the highest satisfaction category (“very satisfied” or “to a very great extent”), according to the wording of each questionnaire item. Values are presented with corresponding 95% confidence intervals (95% CI). The *p*-values correspond to Monte Carlo simulations of the Pearson Chi-square test. Statistical significance was defined as *p* < 0.05.

**Table 4:** Cost efficiency estimated using Stochastic Frontier Analysis (SFA).

Hospital Type	Mean Inefficiency Score	Estimated Cost Efficiency	Interpretation
General hospital	0.253	0.747	Higher cost efficiency
Monospecialty hospital	0.395	0.605	Lower cost efficiency
Difference between hospital types	0.142	—	*p* < 0.001

Note: Cost efficiency was estimated using a stochastic frontier regression model. Lower inefficiency scores indicate higher cost efficiency. Estimated cost efficiency was calculated as 1 − inefficiency score for descriptive presentation.

## Data Availability

The data presented in this study are available from the corresponding authors upon reasonable request. The data are not publicly available due to ethical restrictions and the need to preserve the confidentiality of the participating institutions and study participants.

## Supplementary Materials

The following supporting information can be downloaded at: https://www.mdpi.com/article/10.3390/healthcare14172671/s1, Table S1: Patient Satisfaction Questionnaire Used in the Study.
