# Supplementary material for: An Integrated Multidimensional Framework for Hospital Performance Assessment: Design and Application in Romanian Public Hospitals
Source: Healthcare (Basel). 2026 Aug 22;14(17):2671. doi: 10.3390/healthcare14172671 (PMC13565160; doi:10.3390/healthcare14172671)
Supplement: Supplementary file 1 [file healthcare-14-02671-s001.zip › healthcare-4500050-supplementary.pdf]

Table S1. Patient satisfaction questionnaire used in the study.

No.	Questionnaire item	Response scale
1	Overall satisfaction with the quality of medical services received in the hospital	Very satisfied / Satisfied / Neither satisfied nor dissatisfied / Dissatisfied / Very dissatisfied
2	Satisfaction with the way patient rights were respected	Very satisfied / Satisfied / Neither satisfied nor dissatisfied / Dissatisfied / Very dissatisfied
3	Satisfaction with the waiting time until the first examination by a physician after admission	Very satisfied / Satisfied / Neither satisfied nor dissatisfied / Dissatisfied / Very dissatisfied
4	Satisfaction with the consultation provided by the attending physician	Very satisfied / Satisfied / Neither satisfied nor dissatisfied / Dissatisfied / Very dissatisfied
5	Satisfaction with the treatment received during hospitalization	Very satisfied / Satisfied / Neither satisfied nor dissatisfied / Dissatisfied / Very dissatisfied
6	The attending physician treated the patient with appropriate respect and attention	To a very great extent / To a great extent / To a moderate extent / To a small extent / To a very small extent
7	The attending physician answered the patient's questions	To a very great extent / To a great extent / To a moderate extent / To a small extent / To a very small extent
8	The attending physician carefully addressed the patient's case	To a very great extent / To a great extent / To a moderate extent / To a small extent / To a very small extent
9	The attending physician understood the patient's medical problems	To a very great extent / To a great extent / To a moderate extent / To a small extent / To a very small extent
10	The attending physician showed interest in resolving the patient's medical problem	To a very great extent / To a great extent / To a moderate extent / To a small extent / To a very small extent
11	Nursing staff treated the patient with appropriate respect and attention	To a very great extent / To a great extent / To a moderate extent / To a small extent / To a very small extent
12	Nursing staff answered the patient's questions	To a very great extent / To a great extent / To a moderate extent / To a small extent / To a very small extent
13	Nursing staff respected the prescribed medication administration schedule	To a very great extent / To a great extent / To a moderate extent / To a small extent / To a very small extent
14	Nursing staff understood the patient's medical problems	To a very great extent / To a great extent / To a moderate extent / To a small extent / To a very small extent
15	Nursing staff showed interest in the patient's care	To a very great extent / To a great extent / To a moderate extent / To a small extent / To a very small extent
16	Information and explanations provided by healthcare staff regarding how the medical examination would be performed	To a very great extent / To a great extent / To a moderate extent / To a small extent / To a very small extent

17	Information and explanations provided by healthcare staff regarding the collection of blood or other biological samples	To a very great extent / To a great extent / To a moderate extent / To a small extent / To a very small extent
18	Information and explanations provided by healthcare staff regarding medication administration	To a very great extent / To a great extent / To a moderate extent / To a small extent / To a very small extent

Note: Responses were recorded using five-point Likert-type scales, with lower scores indicating higher levels of satisfaction or more favorable patient perceptions, as applicable.
